# A Guide to the Clinical Examination of the Blood for Diagnostic Purposes

**Published:** 1901-12

**Authors:** 


A GUIDE TO THE CLINICAL EXAMINATION OF THE BLOOD FOR DIAGNOSTIC PURPOSES. By RICHARD C. CABOT, M. D. With colored plates and engravings. Fourth revised edition. Octavo, pp. 515. New York: William Wood & Company. 1901.

When the first edition of this book appeared, four years ago, it was the only one of its kind in English. Hematology was then a new subject, the value of systematic blood examinations just beginning to be understood. Now in many cases blood examination is an essential, and with the simplification in method which time and thought have brought about, a knowledge of certain conditions may be determined as easily as the estimation of urea in urine and should be done as often.

The fourth edition has been almost entirely rewritten. Many changes appear, most noticably in the chapters on typhoid fever, leukemia and diseases due to animal parasites.

The author has quoted freely from all recognised authorities on diseases of the blood, yet in the main the book is based on observations in the Massachusetts General Hospital, 12,000 in all.

A hematologist may perhaps be critical in discussing this work. A general practitioner interested in practical blood examinations as they bear upon medical and surgical cases, finds it most satisfactory. M. J. F.

